# 3*H*-1,2-Benzoxaphosphepine 2-oxides as selective inhibitors of carbonic anhydrase IX and XII

**DOI:** 10.1080/14756366.2022.2143496

**Published:** 2022-11-14

**Authors:** Aleksandrs Pustenko, Anastasija Balašova, Alessio Nocentini, Claudiu T. Supuran, Raivis Žalubovskis

**Affiliations:** a Latvian Institute of Organic Synthesis, Riga, Latvia; b Institute of Technology of Organic Chemistry, Faculty of Materials Science and Applied Chemistry, Riga Technical University, Riga, Latvia; c Department of Neurofarba, Università degli Studi di Firenze, Florence, Italy

**Keywords:** Carbonic anhydrase, benzoxaphosphepine 2-oxide, isoform-selective inhibitors, anti-tumour

## Abstract

The synthesis of 3*H*-1,2-benzoxaphosphepine 2-oxides and evaluation of their inhibitory activity against human carbonic anhydrase (hCA) isoforms I, II, IX, and XII are described. The target compounds were obtained via a concise synthesis from commercial salicylaldehydes and displayed low to sub-micromolar inhibition levels against the tumour-associated isoforms hCA IX and XII. All obtained benzoxaphosphepine 2-oxides possess remarkable selectivity for inhibition of hCA IX/XII over the off-target cytosolic hCA isoforms I and II, whose inhibition may lead to side effects.

## Graphical Abstract

**Figure:**
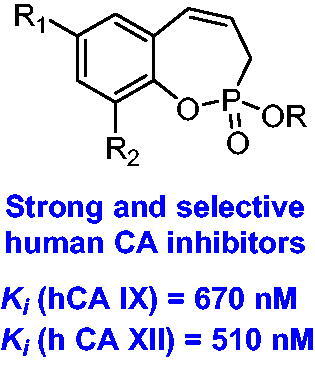

## Introduction

Carbonic anhydrases (CA, EC 4.2.1.1) are a superfamily of metalloenzymes present across all kingdoms of life^1^. These enzymes catalyse a simple yet essential physiological reaction – the reversible hydration of carbon dioxide^2^. To date, 15 different human CA (hCA) isoforms have been identified, out of which hCA IX and XII isoforms are highly overexpressed in different tumour types and may contribute in the survival and progression of tumour cells by regulating intra- and extracellular pH^2–6^. Therefore, the development of selective hCA IX/XII inhibitors is a potential strategy for designing anti-tumour agents.

Due to the high degree of structural homology and sequence similarities within the active site of the hCA isoforms, the design and development of isoform-selective hCA inhibitors pose a challenge^7^. A variety of compounds have been reported as potent and selective inhibitors of tumour-associated isoforms hCA IX and XII including coumarins^8–11^, thiocoumarins^8^^,^^11^, sulphocoumarins^8^^,^^12–15^, as well as their congeners, homosulphocoumarins (3*H*-1,2-benzoxathiepine 2,2-dioxides)^16^. In this work, attention was drawn to phosphorus, as phosphorus-containing molecules display a multitude of biological activities relevant in medicinal chemistry^17^. Additionally, several groups have shown the use of organophosphorus compounds as CA inhibitors^18^.

Considering isosteric relationship between sulphonyl derivatives and phosphonates^19^, our research group designed and synthesised a series of benzoxaphosphepine 2-oxides pursuing the development of new classes of selective CA inhibitors. These compounds showed interesting inhibitory activity against hCA IX and XII. Moreover, the results of current study demonstrate the bioisosteric utility of the cyclic phosphonate moiety in the design of novel CA inhibitors.

## Materials and methods

### Chemistry

The air- or moisture-sensitive reactions were performed under argon atmosphere using dry glassware. Toluene was freshly distilled from Na prior to use. DCM and NEt_3_ were distilled from CaH_2_. Other reagents, starting materials and solvents were purchased from commercial sources and used as received. TLC was performed on silica gel plates (60 F_254_) and visualised under UV light (254 and 365 nm). Melting points were determined on an OptiMelt MPA100 apparatus. IR spectra were recorded on a Shimadzu FTIR IR Prestige-21 spectrophotometer. ^1^H, ^13^C, and ^31^P NMR spectra were recorded on a Bruker Avance Neo 400 MHz spectrometer. The chemical shifts (δ) were reported in parts per million (ppm) relative to the residual solvent peak as an internal reference (DMSO-*d*_6_: ^1^H 2.50, ^13^C 39.52; CDCl_3_: ^1^H 7.26, ^13^C 77.16; C_6_D_6_: ^1^H 7.16, ^13^C 128.06). ^31^P shifts were referenced externally to H_3_PO_4_. The coupling constants (*J*) were expressed in Hertz (Hz). HRMS was performed on a Q-TOF Micro mass spectrometer.

### General procedure for the synthesis of vinylphenols 2

To a stirred solution of MePPh_3_Br (2.3 eq) in dry THF (3 ml/1 mmol of MePPh_3_Br) was added *t*-BuOK (2.35 eq) in small portions over 20 min. The reaction mixture was stirred under inert atmosphere for 2 h at rt. The corresponding hydroxybenzaldehyde **1** (1.0 eq) was added, and the mixture was kept stirring at rt for another 18 h. The reaction mixture was treated with sat. aq NH_4_Cl (25 ml) and then was diluted with Et_2_O (3 ml/1 mmol of MePPh_3_Br). The organic layer was washed with water (2 × 40 ml) and brine (2 × 40 ml), dried over Na_2_SO_4_, filtered, and concentrated *in vacuo*. The crude product was purified by column chromatography on silica gel (PE/EtOAc 4:1).

#### 2-Vinylphenol (2a)


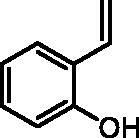
By following the general procedure, **2a** was prepared from MePPh_3_Br (13.46 g, 37.7 mmol), *t*-BuOK (4.32 g, 38.5 mmol), and 2-hydroxybenzaldehyde (**1a**) (2.00 g, 16.4 mmol) as a yellowish oil (1.71 g, 87%)^16^^a^. ^1^H NMR (400 MHz, CDCl_3_) δ = 5.34–5.38 (m, 2H), 5.75 (dd, 1H, *J* = 17.8, 1.4 Hz), 6.80 (dd, 1H, *J* = 8.1, 1.1 Hz), 6.89–7.01 (m, 2H), 7.12–7.17 (m, 1H), 7.40 (dd, 1H, *J* = 7.7, 1.7 Hz) ppm. ^13^C NMR (101 MHz, CDCl_3_) δ = 115.8, 116.0, 121.0, 125.0, 127.4, 129.0, 131.6, 153.0 ppm.

#### 4-Iodo-2-vinylphenol (2b)


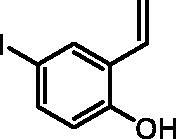
By following the general procedure, **2b** was prepared from MePPh_3_Br (16.56 g, 46.4 mmol), *t*-BuOK (5.32 g, 47.4 mmol), and 2-hydroxy-5-iodobenzaldehyde (**1b**) (5.00 g, 20.2 mmol) as a yellowish solid (4.17 g, 84%)^16^^b^. ^1^H NMR (400 MHz, CDCl_3_) δ = 5.23 (dd, 1H, *J* = 11.3, 1.3 Hz), 5.80 (dd, 1H, *J* = 17.6, 1.3 Hz), 6.67 (d, 1H, *J* = 8.6 Hz), 6.77–6.87 (m, 1H), 7.37 (dd, 1H, *J* = 8.6, 2.4 Hz), 7.70 (d, 1H, *J* = 2.4 Hz), 9.94 (s, 1H) ppm. ^13^C NMR (101 MHz, CDCl_3_) δ = 81.4, 115.1, 118.4, 126.9, 130.4, 134.4, 137.0, 154.6 ppm.

#### 4-Bromo-2-vinylphenol (2c)


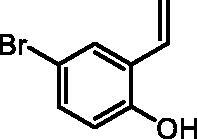
By following the general procedure, **2c** was prepared from MePPh_3_Br (8.17 g, 22.9 mmol), *t*-BuOK (2.62 g, 23.4 mmol), and 5-bromo-2-hydroxybenzaldehyde (**1c**) (2.00 g, 10 mmol) as a yellowish solid (1.74 g, 88%)^16^^a^. ^1^H NMR (400 MHz, CDCl_3_) δ = 4.98 (s, 1H), 5.40 (dd, 1H, *J* = 11.3, 1.0 Hz), 5.74 (dd, 1H, *J* = 17.8, 1.0 Hz), 6.68 (d, 1H, *J* = 8.6 Hz), 6.85 (dd, 1H, *J* = 17.8, 11.3 Hz), 7.23 (dd, 1H, *J* = 8.6, 2.4 Hz), 7.49 (d, 1H, *J* = 2.4 Hz) ppm.

#### 2-Bromo-6-vinylphenol (2d)


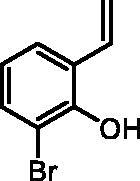
By following the general procedure, **2d** was prepared from MePPh_3_Br (16.35 g, 45.8 mmol), *t*-BuOK (5.25 g, 46.8 mmol), and 3-bromo-2-hydroxybenzaldehyde (**1d**) (4.00 g, 19.9 mmol) as a yellowish solid (3.05 g, 77%)^16^^b^. ^1^H NMR (400 MHz, CDCl_3_) δ = 5.34 (dd, 1H, *J* = 11.2, 1.3 Hz), 5.72 (s, 1H), 5.79 (dd, 1H, *J* = 17.7, 1.3 Hz), 6.77–6.82 (m, 1H), 7.00 (dd, 1H, *J* = 17.7, 11.2 Hz), 7.35–7.41 (m, 2H) ppm. ^13^C NMR (101 MHz, CDCl_3_) δ = 111.2, 116.2, 121.6, 126.2, 126.5, 131.1, 131.3, 149.6 ppm.

#### 2-Methoxy-6-vinylphenol (2e)


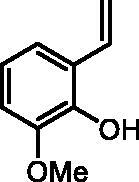
By following the general procedure, **2e** was prepared from MePPh_3_Br (2.70 g, 7.6 mmol), *t*-BuOK (0.87 g, 7.7 mmol), and 2-hydroxy-3-methoxybenzaldehyde (**1e**) (0.50 g, 3.3 mmol) as a yellowish solid (0.40 g, 81%)^20^. ^1^H NMR (400 MHz, CDCl_3_) δ = 3.89 (s, 3H), 5.33 (dd, 1H, *J* = 11.2, 1.5 Hz), 5.83 (dd, 1H, *J* = 17.8, 1.5 Hz), 5.93–5.94 (m, 1H), 6.76–6.80 (m, 1H), 6.81–6.86 (m, 1H), 7.00–7.11 (m, 2H) ppm. ^13^C NMR (101 MHz, CDCl_3_) δ = 56.2, 109.7, 114.9, 118.9, 119.5, 124.1, 131.2, 143.4, 146.8 ppm.

#### 2,4-Dichloro-6-vinylphenol (2f)


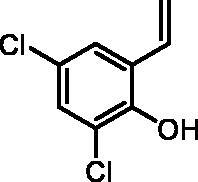
By following the general procedure, **2f** was prepared from MePPh_3_Br (2.15 g, 6.0 mmol), *t*-BuOK (0.69 g, 6.2 mmol), and 3,5-dichloro-2-hydroxybenzaldehyde (**1f**) (0.50 g, 2.6 mmol) as a yellowish solid (0.38 g, 76%)^21^. ^1^H NMR (400 MHz, CDCl_3_) δ = 5.39 (dd, 1H, *J* = 11.2, 1.0 Hz), 5.70–5.72 (m, 1H), 5.80 (d, 1H, *J* = 17.7, 1.0 Hz), 6.92 (dd, 1H, *J* = 17.7, 11.2 Hz), 7.23 (d, 1H, *J* = 2.5 Hz), 7.32–7.34 (m, 1H) ppm. ^13^C NMR (101 MHz, CDCl_3_) δ = 117.3, 121.0, 125.5, 125.6, 127.2, 127.4, 130.1, 147.5 ppm.

#### 4-Nitro-2-vinylphenol (2g)


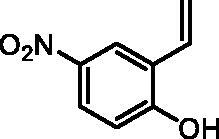
By following the general procedure, **2g** was prepared from MePPh_3_Br (9.83 g, 27.5 mmol), *t*-BuOK (3.16 g, 28.1 mmol), and 2-hydroxy-5-nitrobenzaldehyde (**1g**) (2.00 g, 11 mmol). The solution of nitrobenzaldehyde **1g** in THF (20 ml) was added at −78 °C. The title product was obtained as a yellow solid (1.70 g, 86%)^22^. ^1^H NMR (400 MHz, DMSO-*d_6_*) δ = 5.39 (dd, 1H, *J* = 11.3, 1.2 Hz), 5.98 (dd, 1H, *J* = 17.8, 1.2 Hz), 6.92 (dd, 1H, *J* = 17.8, 11.3 Hz), 7.01 (d, 1H, *J* = 9.0 Hz), 8.02 (dd, 1H, *J* = 9.0, 2.9 Hz), 8.28 (d, 1H, *J* = 2.9 Hz), 11.32 (s, 1H) ppm. ^13^C NMR (101 MHz, DMSO-*d_6_*) δ = 116.1, 116.9, 122.3, 124.7, 124.8, 130.1, 139.9, 161.0 ppm.

#### Diethyl allylphosphonate (S1)


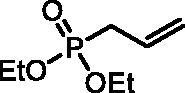
Triethylphosphite (31.0 ml, 180.5 mmol) and allyl bromide (18.7 ml, 216.7 mmol) were stirred and heated for 24 h at 70 °C. After evaporation of the remaining allyl bromide, the residue was distilled *in vacuo* (∼4 mbar) to afford product **S1** as a colourless liquid (29.00 g, 90%), b.p. 60–62 °C/4 mbar^23^. ^31^P NMR (162 MHz, DMSO-*d_6_*) δ = 26.81 ppm. ^1^H NMR (400 MHz, DMSO-*d_6_*) δ = 1.22 (t, 6H, *J* = 7.1 Hz), 2.62 (dt, 1H, *J* = 7.3, 1.3 Hz), 2.67 (dt, 1H, *J* = 7.3, 1.3 Hz), 3.93–4.06 (m, 4H), 5.10–5.26 (m, 2H), 5.63–5.73 (m, 1H) ppm. ^13^C NMR (101 MHz, DMSO-*d_6_*) δ = 16.2 (d, *J*_P,C_ = 5.7 Hz), 30.6 (d, *J*_P,C_ = 136 Hz), 61.2 (d, *J*_P,C_ = 6.3 Hz), 119.5 (d, *J*_P,C_ = 14.2 Hz), 128.3 (d, *J*_P,C_ = 10.9 Hz) ppm.

#### Ethyl allylphosphonochloridate (3)


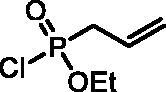
Diethyl allylphosphonate(**S1**) (28.6 g, 160.5 mmol) was dissolved in dry DCM (200 ml). The solution was cooled down to 0 °C, and oxalyl chloride (49.0 ml, 0.562 mol) was added dropwise. The reaction mixture was stirred for 16 h at rt. After evaporation of the solvent and remaining oxalyl chloride, the residue was distilled *in vacuo* (∼4 mbar) to afford product **3** as a colourless liquid (21.64 g, 80%), b.p. 88–90 °C/4 mbar^23^. ^31^P NMR (162 MHz, DMSO-*d_6_*) δ = 39.17 ppm. ^1^H NMR (400 MHz, DMSO-*d_6_*) δ = 1.37 (t, 3H, *J* = 7.1 Hz), 2.93 (dt, 1H, *J* = 7.3, 1.2 Hz), 2.99 (dt, 1H, *J* = 7.3, 1.2 Hz), 4.16–4.37 (m, 2H), 5.26–5.36 (m, 2H), 5.72–5.86 (m, 1H) ppm. ^13^C NMR (101 MHz, DMSO-*d_6_*) δ = 15.9 (d, *J*_P,C_ = 7.0 Hz), 39.1 (d, *J*_P,C_ = 123 Hz), 63.5 (d, *J*_P,C_ = 8.4 Hz), 122.2 (d, *J*_P,C_ = 16.8 Hz), 125.4 (d, *J*_P,C_ = 12.8 Hz) ppm.

### General procedure for the synthesis of diolefins 4

The corresponding vinylphenol **2** (1.0 eq) was dissolved in dry DCM (10 ml/1 mmol of **2**). After cooling down the solution to 0 °C, ethyl allylphosphonochloridate (**3**) (1.2 eq) and NEt_3_ (1.25 eq) were added. The reaction mixture was stirred under inert atmosphere at rt for 18 h. Water (30 ml) was added, and the mixture was extracted with EtOAc (3 × 40 ml). The combined organic layers were washed with brine (2 × 40 ml), dried over Na_2_SO_4_, filtered, and concentrated *in vacuo*. The crude product was purified by column chromatography on silica gel (PE/EtOAc 1.5:1).

#### Ethyl (2-vinylphenyl) allylphosphonate (4a)


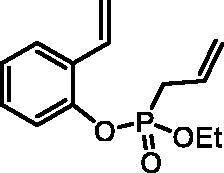
By following the general procedure, **4a** was prepared from 2-vinylphenol (**2a**) (0.38 g, 3.16 mmol), ethyl allylphosphonochloridate (**3**) (0.56 ml, 3.79 mmol), and NEt_3_ (0.55 ml, 3.95 mmol) as a colourless oil (0.48 g, 60%). IR (thin film, cm^−1^): 1260 (P = O), 1219 (P = O). ^31^P NMR (162 MHz, DMSO-*d_6_*) δ = 24.63 ppm. ^1^H NMR (400 MHz, DMSO-*d_6_*) δ = 1.19 (t, 3H, *J* = 7.0 Hz), 2.84–2.97 (m, 2H), 4.00–4.16 (m, 2H), 5.19–5.32 (m, 2H), 5.36 (dd, 1H, *J* = 11.2, 0.9 Hz), 5.71–5.83 (m, 1H), 5.86 (dd, 1H, *J* = 17.7, 0.9 Hz), 6.96 (dd, 1H, *J* = 11.7, 11.2 Hz), 7.16–7.22 (m, 1H), 7.26–7.33 (m, 2H), 7.67 (d, 1H, *J* = 7.7 Hz) ppm. ^13^C NMR (101 MHz, DMSO-*d_6_*) δ = 16.1 (d, *J*_P,C_ = 5.8 Hz), 31.0 (d, *J*_P,C_ = 138 Hz), 62.6 (d, *J*_P,C_ = 6.8 Hz), 116.3, 120.4 (d, *J*_P,C_ = 14.6 Hz), 120.9 (d, *J*_P,C_ = 2.8 Hz), 125.0, 126.3, 127.5 (d, *J*_P,C_ = 11.4 Hz), 128.8 (d, *J*_P,C_ = 5.0 Hz), 129.1, 130.2, 147.4 (d, *J*_P,C_ = 8.9 Hz) ppm. HRMS (ESI) [M + H]^+^: *m/z* calcd for C_13_H_18_O_3_P: 253.0994, found 253.1003.

#### Ethyl (4-iodo-2-vinylphenyl) allylphosphonate (4b)


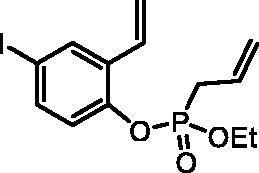
By following the general procedure, **4b** was prepared from 4-iodo-2-vinylphenol (**2b**) (2.50 g, 10.2 mmol), ethyl allylphosphonochloridate (**3**) (1.81 ml, 12.2 mmol), and NEt_3_ (1.77 ml, 12.7 mmol) as a colourless oil (3.53 g, 92%). IR (thin film, cm^−1^): 1265 (P = O), 1220 (P = O). ^31^P NMR (162 MHz, DMSO-*d_6_*) δ = 25.01 ppm. ^1^H NMR (400 MHz, DMSO-*d_6_*) δ = 1.19 (t, 3H, *J* = 7.1 Hz), 2.90 (dt, 1H, *J* = 7.3, 1.2 Hz), 2.96 (dt, 1H, *J* = 7.3, 1.2 Hz), 4.00–4.16 (m, 2H), 5.19–5.32 (m, 2H), 5.40 (dd, 1H, *J* = 11.2, 0.7 Hz), 5.69–5.83 (m, 1H), 5.93 (dd, 1H, *J* = 17.7, 0.7 Hz), 6.84 (dd, 1H, *J* = 17.7, 11.2 Hz), 7.11 (dd, 1H, *J* = 8.6, 1.3 Hz), 7.63 (dd, 1H, *J* = 8.6, 2.2 Hz), 7.98 (d, 1H, *J* = 2.2 Hz) ppm. ^13^C NMR (101 MHz, DMSO-*d_6_*) δ = 16.1 (d, *J*_P,C_ = 5.6 Hz), 30.9 (d, *J*_P,C_ = 137 Hz), 62.7 (d, *J*_P,C_ = 6.7 Hz), 89.6 (d, *J*_P,C_ = 1.4 Hz), 117.9, 120.5 (d, *J*_P,C_ = 15.0 Hz), 123.2 (d, *J*_P,C_ = 2.7 Hz), 127.3 (d, *J*_P,C_ = 11.6 Hz), 128.8, 131.3 (d, *J*_P,C_ = 5.3 Hz), 132.2 (d, *J*_P,C_ = 2.6 Hz), 134.7, 137.5, 147.3 (d, *J*_P,C_ = 9.0 Hz) ppm. HRMS (ESI) [M + H]^+^: *m/z* calcd for C_13_H_17_O_3_PI: 378.9960, found 378.9966.

#### Ethyl (4-bromo-2-vinylphenyl) allylphosphonate (4c)


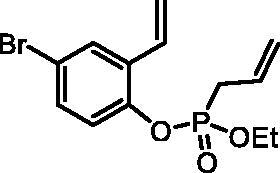
By following the general procedure, **4c** was prepared from 4-bromo-2-vinylphenol (**2c**) (1.63 g, 8.19 mmol), ethyl allylphosphonochloridate (**3**) (1.46 ml, 9.83 mmol), and NEt_3_ (1.42 ml, 10.2 mmol) as a colourless oil (1.71 g, 63%). IR (thin film, cm^−1^): 1266 (P = O), 1224 (P = O), 1174 (P = O). ^31^P NMR (162 MHz, DMSO-*d_6_*) δ = 25.10 ppm. ^1^H NMR (400 MHz, DMSO-*d_6_*) δ = 1.16–1.22 (m, 3H), 2.91 (dt, 1H, *J* = 7.3, 1.2 Hz), 2.97 (dt, 1H, *J* = 7.3, 1.2 Hz), 3.99–4.17 (m, 2H), 5.19–5.32 (m, 2H), 5.43 (dd, 1H, *J* = 11.2, 0.8 Hz), 5.70–5.83 (m, 1H), 5.97 (dd, 1H, *J* = 17.7, 0.8 Hz), 6.87 (dd, 1H, *J* = 17.7, 11.2 Hz), 7.26 (dd, 1H, *J* = 8.7, 1.3 Hz), 7.48 (dd, 1H, *J* = 8.7, 2.5 Hz), 7.86 (d, 1H, *J* = 2.5 Hz) ppm. ^13^C NMR (101 MHz, DMSO-*d_6_*) δ = 16.1 (d, *J*_P,C_ = 6.0 Hz), 30.9 (d, *J*_P,C_ = 137 Hz), 62.8 (d, *J*_P,C_ = 6.7 Hz), 117.3 (d, *J*_P,C_ = 1.5 Hz), 118.2, 120.5 (d, *J*_P,C_ = 15.0 Hz), 123.0 (d, *J*_P,C_ = 2.8 Hz), 127.3 (d, *J*_P,C_ = 11.8 Hz), 128.8 (d, *J*_P,C_ = 9.7 Hz), 131.1 (d, *J*_P,C_ = 5.3 Hz), 131.6, 146.6 (d, *J*_P,C_ = 8.9 Hz) ppm. HRMS (ESI) [M + H]^+^: *m/z* calcd for C_13_H_17_O_3_PBr: 331.0099, found 331.0103.

#### 2-Bromo-6-vinylphenyl ethyl allylphosphonate (4d)


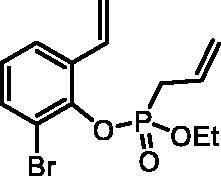
By following the general procedure, **4d** was prepared from 2-bromo-6-vinylphenol (**2d**) (1.00 g, 5.02 mmol), ethyl allylphosphonochloridate (**3**) (0.89 ml, 6.03 mmol), and NEt_3_ (0.87 ml, 6.28 mmol) as a colourless oil (1.28 g, 77%). IR (thin film, cm^−1^): 1262 (P = O), 1219 (P = O). ^31^P NMR (162 MHz, DMSO-*d_6_*) δ = 25.07 ppm. ^1^H NMR (400 MHz, DMSO-*d_6_*) δ = 1.16 (dt, 3H, *J* = 7.0, 0.4 Hz), 3.00–3.10 (m, 2H), 3.96–4.15 (m, 2H), 5.22–5.37 (m, 2H), 5.42 (dd, 1H, *J* = 11.1, 0.9 Hz), 5.77–5.92 (m, 2H), 7.06 (dd, 1H, *J* = 17.7, 11.1 Hz), 7.13–7.19 (m, 1H), 7.61 (dd, 1H, *J* = 7.9, 1.5 Hz), 7.69 (dd, 1H, *J* = 7.9, 1.5 Hz) ppm. ^13^C NMR (101 MHz, DMSO-*d_6_*) δ = 16.0 (d, *J*_P,C_ = 6.0 Hz), 32.1 (d, *J*_P,C_ = 139 Hz), 63.1 (d, *J*_P,C_ = 6.9 Hz), 116.5 (d, *J*_P,C_ = 4.0 Hz), 117.6, 120.5 (d, *J*_P,C_ = 15.2 Hz), 125.6 (d, *J*_P,C_ = 1.5 Hz), 126.7, 127.4 (d, *J*_P,C_ = 11.4 Hz), 130.7, 132.2 (d, *J*_P,C_ = 2.8 Hz), 132.8 (d, *J*_P,C_ = 1.5 Hz), 145.0 (d, *J*_P,C_ = 10.7 Hz) ppm. HRMS (ESI) [M + H]^+^: *m/z* calcd for C_13_H_17_O_3_PBr: 331.0099, found 331.0092.

#### Ethyl (2-methoxy-6-vinylphenyl) allylphosphonate (4e)


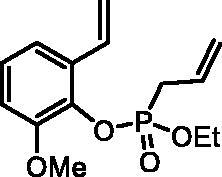
By following the general procedure, **4e** was prepared from 2-methoxy-6-vinylphenol (**2e**) (0.32 g, 2.13 mmol), ethyl allylphosphonochloridate (**3**) (0.38 ml, 2.56 mmol), and NEt_3_ (0.37 ml, 2.66 mmol) as a colourless oil (0.40 g, 66%). IR (thin film, cm^−1^): 1274 (P = O), 1179 (P = O). ^31^P NMR (162 MHz, DMSO-*d_6_*) δ = 24.86 ppm. ^1^H NMR (400 MHz, DMSO-*d_6_*) δ = 1.19–1.24 (m, 3H), 2.86–2.96 (m, 2H), 3.82 (s, 3H), 3.96–4.18 (m, 2H), 5.18–5.31 (m, 2H), 5.37 (dd, 1H, *J* = 11.1, 1.1 Hz), 5.74–5.88 (m, 2H), 6.97 (dd, 1H, *J* = 17.7, 11.1 Hz), 7.04 (dd, 1H, *J* = 8.1, 1.5 Hz), 7.11–7.17 (m, 1H), 7.22 (dd, 1H, *J* = 7.9, 1.5 Hz) ppm. ^13^C NMR (101 MHz, DMSO-*d_6_*) δ = 16.2 (d, *J*_P,C_ = 6.1 Hz), 31.8 (d, *J*_P,C_ = 139 Hz), 55.9, 62.1 (d, *J*_P,C_ = 7.0 Hz), 112.2, 116.6, 117.3, 119.9 (d, *J*_P,C_ = 15.0 Hz), 125.2, 128.1 (d, *J*_P,C_ = 11.5 Hz), 130.4, 130.5 (d, *J*_P,C_ = 3.5 Hz), 137.1 (d, *J*_P,C_ = 9.6 Hz), 151.2 (d, *J*_P,C_ = 3.0 Hz) ppm. HRMS (ESI) [M + H]^+^: *m/z* calcd for C_14_H_20_O_4_P: 283.1099, found 283.1105.

#### 2,4-Dichloro-6-vinylphenyl ethyl allylphosphonate (4f)


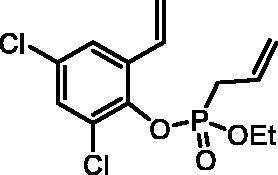
By following the general procedure, **4f** was prepared from 2,4-dichloro-6-vinylphenol (**2f**) (0.80 g, 4.23 mmol), ethyl allylphosphonochloridate (**3**) (0.75 ml, 5.08 mmol), and NEt_3_ (0.74 ml, 5.29 mmol) as a colourless oil (1.17 g, 86%). IR (thin film, cm^−1^): 1262 (P = O), 1217 (P = O). ^31^P NMR (162 MHz, C_6_D_6_) δ = 24.33 ppm. ^1^H NMR (400 MHz, C_6_D_6_) δ = 0.88 (dt, 3H, *J* = 7.1, 0.4 Hz), 2.63–2.73 (m, 2H), 3.76–3.98 (m, 2H), 5.00–5.11 (m, 3H), 5.27 (d, 1H, *J* = 17.6 Hz), 5.76–5.89 (m, 1H), 7.03 (d, 1H, *J* = 2.5 Hz), 7.19–7.21 (m, 1H), 7.25 (dd, 1H, *J* = 17.6, 11.0 Hz) ppm. ^13^C NMR (101 MHz, C_6_D_6_) δ = 16.3 (d, *J*_P,C_ = 5.7 Hz), 33.0 (d, *J*_P,C_ = 141 Hz), 63.4 (d, *J*_P,C_ = 7.0 Hz), 118.0, 120.4 (d, *J*_P,C_ = 15.0 Hz), 125.0 (d, *J*_P,C_ = 1.9 Hz), 127.5 (d, *J*_P,C_ = 11.5 Hz), 128.9 (d, *J*_P,C_ = 3.7 Hz), 129.4, 130.8, 130.9 (d, *J*_P,C_ = 2.1 Hz), 134.3 (d, *J*_P,C_ = 3.0 Hz), 143.8 (d, *J*_P,C_ = 10.7 Hz) ppm. HRMS (ESI) [M + H]^+^: *m/z* calcd for C_13_H_16_O_3_PCl_2_: 321.0214, found 321.0233.

#### Ethyl (4-nitro-2-vinylphenyl) allylphosphonate (4 g)


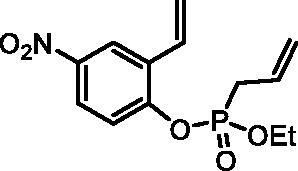
By following the general procedure, **4 g** was prepared from 4-nitro-2-vinylphenol (**2 g**) (1.00 g, 6.06 mmol), ethyl allylphosphonochloridate (**3**) (1.08 ml, 7.27 mmol), and NEt_3_ (1.05 ml, 7.57 mmol) as a yellowish oil (1.70 g, 94%). IR (thin film, cm^−1^): 1273 (P = O), 1232 (P = O). ^31^P NMR (162 MHz, DMSO-*d_6_*) δ = 25.61 ppm. ^1^H NMR (400 MHz, DMSO-*d_6_*) δ = 1.22 (dt, 3H, *J* = 7.1, 0.3 Hz), 3.00 (dt, 1H, *J* = 7.3, 1.2 Hz), 3.06 (dt, 1H, *J* = 7.3, 1.2 Hz), 4.06–4.22 (m, 2H), 5.21–5.35 (m, 2H), 5.54–5.58 (m, 1H), 5.71–5.85 (m, 1H), 6.11 (dd, 1H, *J* = 17.7, 0.6 Hz), 6.96 (dd, 1H, *J* = 17.7, 11.2 Hz), 7.59 (dd, 1H, *J* = 9.1, 1.2 Hz), 8.19 (dd, 1H, *J* = 9.1, 2.9 Hz), 8.45–8.48 (m, 1H) ppm. ^13^C NMR (101 MHz, DMSO-*d_6_*) δ = 16.1 (d, *J*_P,C_ = 5.8 Hz), 30.9 (d, *J*_P,C_ = 137 Hz), 63.1 (d, *J*_P,C_ = 6.9 Hz), 119.6, 120.8 (d, *J*_P,C_ = 15.0 Hz), 121.7 (d, *J*_P,C_ = 3.0 Hz), 121.8, 124.2, 127.0 (d, *J*_P,C_ = 11.7 Hz), 128.6, 129.9 (d, *J*_P,C_ = 5.5 Hz), 144.3, 152.0 (d, *J*_P,C_ = 8.6 Hz) ppm. HRMS (ESI) [M + H]^+^: *m/z* calcd for C_13_H_17_NO_5_P: 298.0844, found 298.0858.

### General procedure for the synthesis of 2-ethoxy-3H-1,2-benzoxaphosphepine 2-oxides 6

The corresponding diolefin **4** (1.0 eq) was dissolved in dry, degassed PhMe (18 ml/1 mmol of **4**). The solution was sparged with argon followed by addition of ruthenium catalyst **5** (CAS: 254972–49-1) (5 mol%). The reaction mixture was stirred at 70 °C for 4 h, then cooled down to rt, and concentrated *in vacuo*. The residue was purified by column chromatography on silica gel (EtOAc 100%).

#### 2-Ethoxy-3H-benzo[f][1,2]oxaphosphepine 2-oxide (6a)


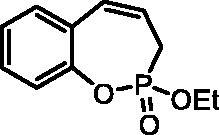
By following the general procedure, **6a** was prepared from ethyl (2-vinylphenyl) allylphosphonate (**4a**) (0.43 g, 1.70 mmol), and ruthenium catalyst **5** (81 mg, 0.085 mmol) as a greenish dense oil (0.28 g, 74%). IR (thin film, cm^−1^): 1265 (P = O), 1203 (P = O). ^31^P NMR (162 MHz, DMSO-*d_6_*) δ = 40.00 ppm. ^1^H NMR (400 MHz, DMSO-*d_6_*) δ = 1.28 (t, 3H, *J* = 7.1 Hz), 2.62–2.88 (m, 2H), 4.14–4.23 (m, 2H), 5.92–6.04 (m, 1H), 6.71 (dd, 1H, *J* = 10.8, 5.3 Hz), 7.17–7.27 (m, 2H), 7.31–7.41 (m, 2H) ppm. ^13^C NMR (101 MHz, DMSO-*d_6_*) δ = 16.3 (d, *J*_P,C_ = 5.9 Hz), 25.5 (d, *J*_P,C_ = 125 Hz), 62.2 (d, *J*_P,C_ = 6.9 Hz), 121.6 (d, *J*_P,C_ = 3.4 Hz), 122.7 (d, *J*_P,C_ = 12.2 Hz), 125.0, 127.6 (d, *J*_P,C_ = 1.1 Hz), 129.4, 129.5, 129.6 130.6, 147.5 (d, *J*_P,C_ = 8.3 Hz) ppm. HRMS (ESI) [M + H]^+^: *m/z* calcd for C_11_H_14_O_3_P: 225.0681, found 225.0692.

#### 2-Ethoxy-7-iodo-3H-benzo[f][1,2]oxaphosphepine 2-oxide (6b)


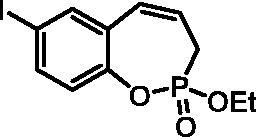
By following the general procedure, **6b** was prepared from ethyl (4-iodo-2-vinylphenyl) allylphosphonate (**4b**) (3.50 g, 9.26 mmol), and ruthenium catalyst **5** (438 mg, 0.46 mmol) as a greenish dense oil (2.46 g, 76%). IR (thin film, cm^−1^): 1265 (P = O), 1173 (P = O). ^31^P NMR (162 MHz, DMSO-*d_6_*) δ = 39.83 ppm. ^1^H NMR (400 MHz, DMSO-*d_6_*) δ = 1.27 (t, 3H, *J* = 7.1 Hz), 2.66–2.92 (m, 2H), 4.13–4.22 (m, 2H), 5.95–6.07 (m, 1H), 6.63–6.70 (m, 1H), 6.99–7.04 (m, 1H), 7.68 (dd, 1H, *J* = 8.5, 2.2 Hz), 7.71 (d, 1H, *J* = 2.2 Hz) ppm. ^13^C NMR (101 MHz, DMSO-*d_6_*) δ = 16.2 (d, *J*_P,C_ = 5.6 Hz), 25.5 (d, *J*_P,C_ = 125 Hz), 62.4 (d, *J*_P,C_ = 6.7 Hz), 89.3 (d, *J*_P,C_ = 1.4 Hz), 123.8, 123.9, 124.0, 128.3 (d, *J*_P,C_ = 8.7 Hz), 130.2, 137.9, 138.8, 147.4 (d, *J*_P,C_ = 8.0 Hz) ppm. HRMS (ESI) [M + H]^+^: *m/z* calcd for C_11_H_13_O_3_PI: 350.9647, found 350.9659.

#### 7-Bromo-2-ethoxy-3H-benzo[f][1,2]oxaphosphepine 2-oxide (6c)


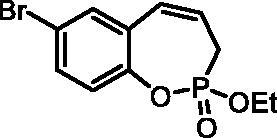
By following the general procedure, **6c** was prepared from ethyl (4-bromo-2-vinylphenyl) allylphosphonate (**4c**) (0.92 g, 2.78 mmol), and ruthenium catalyst **5** (132 mg, 0.14 mmol) as a greenish dense oil (0.53 g, 63%). IR (thin film, cm^−1^): 1274 (P = O), 1220 (P = O). ^31^P NMR (162 MHz, DMSO-*d_6_*) δ = 44.68 ppm. ^1^H NMR (400 MHz, DMSO-*d_6_*) δ = 1.27 (t, 3H, *J* = 7.1 Hz), 2.68–2.93 (m, 2H), 4.13–4.23 (m, 2H), 5.97–6.10 (m, 1H), 6.65–6.71 (m, 1H), 7.15–7.19 (m, 1H), 7.54 (dd, 1H, *J* = 8.6, 2.5 Hz), 7.58 (d, 1H, *J* = 2.5 Hz) ppm. ^13^C NMR (101 MHz, DMSO-*d_6_*) δ = 16.2 (d, *J*_P,C_ = 5.4 Hz), 25.4 (d, *J*_P,C_ = 125 Hz), 62.5 (d, *J*_P,C_ = 7.0 Hz), 116.9 (d, *J*_P,C_ = 1.5 Hz), 123.8 (d, *J*_P,C_ = 3.5 Hz), 124.1 (d, *J*_P,C_ = 12.3 Hz), 128.4 (d, *J*_P,C_ = 9.0 Hz), 130.0, 132.1, 132.9, 146.7 (d, *J*_P,C_ = 7.8 Hz) ppm. HRMS (ESI) [M + H]^+^: *m/z* calcd for C_11_H_13_O_3_PBr: 302.9786, found 302.9791.

#### 9-Bromo-2-ethoxy-3H-benzo[f][1,2]oxaphosphepine 2-oxide (6d)


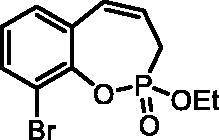
By following the general procedure, **6d** was prepared from 2-bromo-6-vinylphenyl ethyl allylphosphonate (**4d**) (2.00 g, 6.04 mmol), and ruthenium catalyst **5** (286 mg, 0.30 mmol) as a greenish dense oil (1.58 g, 86%). IR (thin film, cm^−1^): 1268 (P = O), 1232 (P = O). ^31^P NMR (162 MHz, DMSO-*d_6_*) δ = 39.08 ppm. ^1^H NMR (400 MHz, DMSO-*d_6_*) δ = 1.29 (t, 3H, *J* = 7.1 Hz), 2.70–2.83 (m, 1H), 2.87–3.00 (m, 1H), 4.18–4.32 (m, 2H), 5.95–6.08 (m, 1H), 6.67–6.74 (m, 1H), 7.17 (td, 1H, *J* = 7.8, 0.6 Hz), 7.34 (dd, 1H, *J* = 7.8, 1.6 Hz), 7.68 (dd, 1H, *J* = 7.8, 1.6 Hz) ppm. ^13^C NMR (101 MHz, DMSO-*d_6_*) δ = 16.2 (d, *J*_P,C_ = 6.1 Hz), 25.8 (d, *J*_P,C_ = 126 Hz), 62.8 (d, *J*_P,C_ = 7.0 Hz), 115.4 (d, *J*_P,C_ = 3.8 Hz), 123.4 (d, *J*_P,C_ = 12.2 Hz), 126.1, 129.1 (d, *J*_P,C_ = 9.0 Hz), 129.3, 130.4, 132.7, 144.2 (d, *J*_P,C_ = 7.8 Hz) ppm. HRMS (ESI) [M + H]^+^: *m/z* calcd for C_11_H_13_O_3_PBr: 302.9786, found 302.9795.

#### 2-Ethoxy-9-methoxy-3H-benzo[f][1,2]oxaphosphepine 2-oxide (6e)


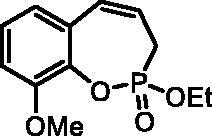
By following the general procedure, **6e** was prepared from ethyl (2-methoxy-6-vinylphenyl) allylphosphonate (**4e**) (315 mg, 1.12 mmol), and ruthenium catalyst **5** (53 mg, 0.056 mmol) as a greenish dense oil (0.23 g, 81%). IR (thin film, cm^−1^): 1270 (P = O), 1244 (P = O). ^31^P NMR (162 MHz, DMSO-*d_6_*) δ = 41.74 ppm. ^1^H NMR (400 MHz, DMSO-*d_6_*) δ = 1.26 (t, 3H, *J* = 7.1 Hz), 2.50–2.63 (m, 1H), 2.79–2.91 (m, 1H), 3.84 (s, 3H), 4.13–4.22 (m, 2H), 5.92–6.04 (m, 1H), 6.65–6.72 (m, 1H), 6.86 (dd, 1H, *J* = 7.8, 1.4 Hz), 7.08 (dd, 1H, *J* = 8.2, 1.4 Hz), 7.14–7.20 (m, 1H) ppm. ^13^C NMR (101 MHz, DMSO-*d_6_*) δ = 16.1 (d, *J*_P,C_ = 6.1 Hz), 25.4 (d, *J*_P,C_ = 127 Hz), 55.9, 62.0 (d, *J*_P,C_ = 6.9 Hz), 112.0, 121.3, 122.9 (d, *J*_P,C_ = 12.2 Hz), 125.0, 128.7, 129.6 (d, *J*_P,C_ = 8.8 Hz), 136.6 (d, *J*_P,C_ = 8.4 Hz), 151.2 (d, *J*_P,C_ = 3.1 Hz) ppm. HRMS (ESI) [M + H]^+^: *m/z* calcd for C_12_H_16_O_4_P: 255.0786, found 255.0800.

#### 7,9-Dichloro-2-ethoxy-3H-benzo[f][1,2]oxaphosphepine 2-oxide (6f)


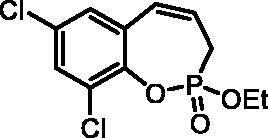
By following the general procedure, **6f** was prepared from 2,4-dichloro-6-vinylphenyl ethyl allylphosphonate (**4f**) (0.70 g, 2.18 mmol), and ruthenium catalyst **5** (103 mg, 0.109 mmol) as a greenish dense oil (0.46 g, 72%). IR (thin film, cm^−1^): 1276 (P = O), 1242 (P = O). ^31^P NMR (162 MHz, DMSO-*d_6_*) δ = 40.02 ppm. ^1^H NMR (400 MHz, DMSO-*d_6_*) δ = 1.29 (t, 3H, *J* = 7.1 Hz), 2.76–3.05 (m, 2H), 4.15–4.31 (m, 2H), 6.02–6.15 (m, 1H), 6.66–6.72 (m, 1H), 7.46 (d, 1H, *J* = 2.6 Hz), 7.73 (d, 1H, *J* = 2.6 Hz) ppm. ^13^C NMR (101 MHz, DMSO-*d_6_*) δ = 16.2 (d, *J*_P,C_ = 6.0 Hz), 25.7 (d, *J*_P,C_ = 126 Hz), 62.9 (d, *J*_P,C_ = 6.9 Hz), 125.0 (d, *J*_P,C_ = 12.2 Hz), 126.8 (d, *J*_P,C_ = 3.6 Hz), 128.0 (d, *J*_P,C_ = 9.2 Hz), 128.8, 128.9, 129.1, 130.8, 142.3 (d, *J*_P,C_ = 7.6 Hz) ppm. HRMS (ESI) [M + H]^+^: *m/z* calcd for C_11_H_12_O_3_PCl_2_: 292.9901, found 292.9908.

#### 2-Ethoxy-7-nitro-3H-benzo[f][1,2]oxaphosphepine 2-oxide (6 g)


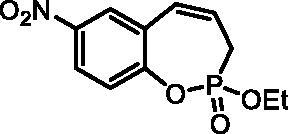
By following the general procedure, **6 g** was prepared from ethyl (4-nitro-2-vinylphenyl) allylphosphonate (**4 g**) (1.85 g, 6.22 mmol), and ruthenium catalyst **5** (295 mg, 0.31 mmol) as a brown dense oil (1.12 g, 67%). IR (thin film, cm^−1^): 1278 (P = O), 1233 (P = O). ^31^P NMR (162 MHz, DMSO-*d_6_*) δ = 38.90 ppm. ^1^H NMR (400 MHz, DMSO-*d_6_*) δ = 1.29 (t, 3H, *J* = 7.1 Hz), 2.78–3.03 (m, 2H), 4.18–4.28 (m, 2H), 6.06–6.19 (m, 1H), 6.81–6.88 (m, 1H), 7.44–7.48 (m, 1H), 8.22 (dd, 1H, *J* = 8.9, 2.8 Hz), 8.30 (d, 1H, *J* = 2.8 Hz) ppm. ^13^C NMR (101 MHz, DMSO-*d_6_*) δ = 16.2 (d, *J*_P,C_ = 5.8 Hz), 25.7 (d, *J*_P,C_ = 124 Hz), 62.8 (d, *J*_P,C_ = 6.8 Hz), 123.2 (d, *J*_P,C_ = 3.8 Hz), 124.6, 125.0 (d, *J*_P,C_ = 12.4 Hz), 126.4, 128.2 (d, *J*_P,C_ = 9.2 Hz), 128.8, 144.1, 151.9 (d, *J*_P,C_ = 8.0 Hz) ppm. HRMS (ESI) [M + H]^+^: *m/z* calcd for C_11_H_13_NO_5_P: 270.0531, found 270.0539.

### General procedure for the synthesis of 2-hydroxy-3H-1,2-benzoxaphosphepine 2-oxides 7

The corresponding ethoxy derivative **6** (1.0 eq) was dissolved in dry DCM (20 ml/1 mmol of **6**), then TMSBr (6.0 eq) was added dropwise. The reaction mixture was stirred under inert atmosphere at rt for 24 h. The volatiles were removed *in vacuo*, and the residue was treated with MeOH (15 ml/1 mmol of **6**), concentrated, purified by column chromatography on silica gel (EtOAc 100%). Products were recrystallised from EtOAc.

#### 2-Hydroxy-3H-benzo[f][1,2]oxaphosphepine 2-oxide(7a)


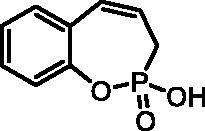
By following the general procedure, **7a** was prepared from 2-ethoxy-3*H*-benzo[*f*][1,2]oxaphosphepine 2-oxide (**6a**) (0.32 g, 1.43 mmol) and TMSBr (1.12 ml, 8.56 mmol) as a white solid (0.25 g, 88%). Mp: 128–129 °C. IR (KBr, cm^−1^): 2487 (O = P-OH), 2203 (O = P-OH), 1665 (O = P-OH), 1258 (P = O), 1223 (P = O). ^31^P NMR (162 MHz, DMSO-*d_6_*) δ = 36.64 ppm. ^1^H NMR (400 MHz, DMSO-*d_6_*) δ = 2.57 (dd, 1H, *J* = 6.7, 1.0 Hz), 2.62 (dd, 1H, *J* = 6.7, 1.0 Hz), 5.88–6.01 (m, 1H), 6.64 (dd, 1H, *J* = 10.8, 5.0 Hz), 7.09–7.14 (m, 1H), 7.17–7.23 (m, 1H), 7.27–7.37 (m, 2H) ppm. ^13^C NMR (101 MHz, DMSO-*d_6_*) δ = 27.1 (d, *J*_P,C_ = 125 Hz), 121.8 (d, *J*_P,C_ = 3.4 Hz), 123.6 (d, *J*_P,C_ = 12.2 Hz), 124.5, 127.9, 129.1 (d, *J*_P,C_ = 8.4 Hz), 129.2, 130.6, 147.9 (d, *J*_P,C_ = 7.6 Hz) ppm. HRMS (ESI) [M + H]^+^: *m/z* calcd for C_9_H_10_O_3_P: 197.0368, found 197.0371.

#### 2-Hydroxy-7-iodo-3H-benzo[f][1,2]oxaphosphepine 2-oxide (7b)


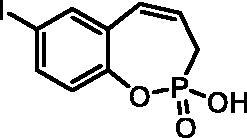
By following the general procedure, **7b** was prepared from 2-ethoxy-7-iodo-3*H*-benzo[*f*][1,2]oxaphosphepine 2-oxide (**6b**) (2.22 g, 6.34 mmol) and TMSBr (4.98 ml, 38.0 mmol) as a white solid (1.66 g, 81%). Mp: 193–194 °C.IR (KBr, cm^−1^): 2490 (O = P-OH), 2198 (O = P-OH), 1652 (O = P-OH), 1259 (P = O), 1217 (P = O). ^31^P NMR (162 MHz, DMSO-*d_6_*) δ = 36.14 ppm. ^1^H NMR (400 MHz, DMSO-*d_6_*) δ = 2.59 (dd, 1H, *J* = 6.7, 1.0 Hz), 2.65 (dd, 1H, *J* = 6.7, 1.0 Hz), 5.91–6.03 (m, 1H), 6.56–6.62 (m, 1H), 6.92 (dd, 1H, *J* = 8.4, 1.1 Hz), 7.64 (dd, 1H, *J* = 8.4, 2.2 Hz), 7.68 (d, 1H, *J* = 2.2 Hz) ppm. ^13^C NMR (101 MHz, DMSO-*d_6_*) δ = 27.1 (d, *J*_P,C_ = 125 Hz), 88.6 (d, *J*_P,C_ = 1.5 Hz), 124.2 (d, *J*_P,C_ = 3.2 Hz), 124.9 (d, *J*_P,C_ = 12.2 Hz), 127.8 (d, *J*_P,C_ = 8.4 Hz), 130.6, 137.6, 138.7, 147.9 (d, *J*_P,C_ = 7.4 Hz) ppm. HRMS (ESI) [M + H]^+^: *m/z* calcd for C_9_H_9_O_3_PI: 322.9334, found 322.9345.

#### 7-Bromo-2-hydroxy-3H-benzo[f][1,2]oxaphosphepine 2-oxide (7c)


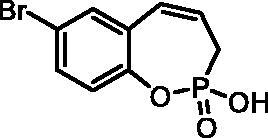
By following the general procedure, **7c** was prepared from 7-bromo-2-ethoxy-3*H*-benzo[*f*][1,2]oxaphosphepine 2-oxide (**6c**) (0.31 g, 1.02 mmol) and TMSBr (0.80 ml, 6.14 mmol) as a white solid (0.23 g, 82%). Mp: 163–164 °C. IR (KBr, cm^−1^): 1652 (O = P-OH), 1224 (P = O), 1206 (P = O). ^31^P NMR (162 MHz, DMSO-*d_6_*) δ = 36.27 ppm. ^1^H NMR (400 MHz, DMSO-*d_6_*) δ = 2.61 (dd, 1H, *J* = 6.7, 0.9 Hz), 2.66 (dd, 1H, *J* = 6.7, 0.9 Hz), 5.93–6.06 (m, 1H), 6.58–6.64 (m, 1H), 7.07 (dd, 1H, *J* = 8.6, 0.9 Hz), 7.50 (dd, 1H, *J* = 8.6, 2.5 Hz), 7.54 (d, 1H, *J* = 2.5 Hz) ppm. ^13^C NMR (101 MHz, DMSO-*d_6_*) δ = 27.1 (d, *J*_P,C_ = 125 Hz), 116.3 (d, *J*_P,C_ = 1.5 Hz), 124.0 (d, *J*_P,C_ = 3.4 Hz), 125.1 (d, *J*_P,C_ = 12.0 Hz), 127.9 (d, *J*_P,C_ = 8.6 Hz), 130.3, 131.8, 132.8, 147.2 (d, *J*_P,C_ = 7.6 Hz) ppm. HRMS (ESI) [M + H]^+^: *m/z* calcd for C_9_H_9_O_3_PBr: 274.9473, found 274.9470.

#### 9-Bromo-2-hydroxy-3H-benzo[f][1,2]oxaphosphepine 2-oxide (7d)


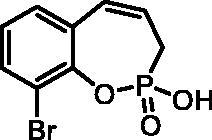
By following the general procedure, **7d** was prepared from 9-bromo-2-ethoxy-3*H*-benzo[*f*][1,2]oxaphosphepine 2-oxide (**6d**) (0.60 g, 1.98 mmol) and TMSBr (1.55 ml, 11.9 mmol) as a white solid (0.49 g, 90%). Mp: 180–181 °C. IR (KBr, cm^−1^): 2545 (O = P-OH), 2125 (O = P-OH), 1214 (P = O), 1210 (P = O). ^31^P NMR (162 MHz, DMSO-*d_6_*) δ = 36.44 ppm. ^1^H NMR (400 MHz, DMSO-*d_6_*) δ = 2.63 (dd, 1H, *J* = 6.6, 0.9 Hz), 2.68 (dd, 1H, *J* = 6.6, 0.9 Hz), 5.92–6.05 (m, 1H), 6.61–6.67 (m, 1H), 7.09–7.14 (m, 1H), 7.30 (dd, 1H, *J* = 7.8, 1.3 Hz), 7.64 (dd, 1H, *J* = 7.9, 1.5 Hz) ppm. ^13^C NMR (101 MHz, DMSO-*d_6_*) δ = 27.3 (d, *J*_P,C_ = 125 Hz), 115.9 (d, *J*_P,C_ = 3.8 Hz), 124.6 (d, *J*_P,C_ = 12.1 Hz), 125.5, 128.6 (d, *J*_P,C_ = 8.6 Hz), 129.7, 130.2, 132.4, 144.9 (d, *J*_P,C_ = 7.4 Hz) ppm. HRMS (ESI) [M + H]^+^: *m/z* calcd for C_9_H_9_O_3_PBr: 274.9473, found 274.9473.

#### 2-Hydroxy-9-methoxy-3H-benzo[f][1,2]oxaphosphepine 2-oxide (7e)


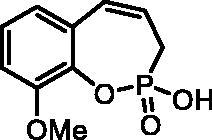
By following the general procedure, **7e** was prepared from 2-ethoxy-9-methoxy-3*H*-benzo[*f*][1,2]oxaphosphepine 2-oxide (**6e**) (185 mg, 0.73 mmol) and TMSBr (0.57 ml, 4.37 mmol) as a white solid (120 mg, 73%). Mp: 200–201 °C. IR (KBr, cm^−1^): 2527 (O = P-OH), 2224 (O=P-OH), 1275 (P = O), 1256 (P = O).^31^P NMR (162 MHz, DMSO-*d_6_*) δ = 37.38 ppm. ^1^H NMR (400 MHz, DMSO-*d_6_*) δ = 2.56 (dd, 1H, *J* = 6.6, 0.9 Hz), 2.61 (dd, 1H, *J* = 6.6, 0.9 Hz), 3.80 (s, 3H), 5.87–5.99 (m, 1H), 6.59 (dd, 1H, *J* = 10.9, 4.8 Hz), 6.82 (dd, 1H, *J* = 7.7, 1.3 Hz), 7.01–7.05 (m, 1H), 7.08–7.14 (m, 1H) ppm. ^13^C NMR (101 MHz, DMSO-*d_6_*) δ = 27.3 (d, *J*_P,C_ = 126 Hz), 55.9, 112.0, 121.6, 123.7 (d, *J*_P,C_ = 12.2 Hz), 124.4, 128.9, 129.0, 129.1, 137.2 (d, *J*_P,C_ = 7.6 Hz), 151.5 (d, *J*_P,C_ = 3.3 Hz) ppm. HRMS (ESI) [M + H]^+^: *m/z* calcd for C_10_H_12_O_4_P: 227.0473, found 227.0477.

#### 7,9-Dichloro-2-hydroxy-3H-benzo[f][1,2]oxaphosphepine 2-oxide (7f)


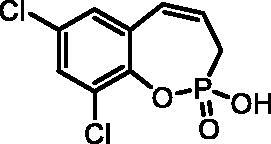


By following the general procedure, **7f** was prepared from 7,9-dichloro-2-ethoxy-3*H*-benzo[*f*][1,2]oxaphosphepine 2-oxide (**6f**) (0.37 g, 1.26 mmol) and TMSBr (1.00 ml, 7.57 mmol) as a white solid (0.27 g, 81%). Mp: 192–193 °C. IR (KBr, cm^−1^): 2522 (O = P-OH), 2219 (O = P-OH), 1230 (P = O), 1155 (P = O).^31^P NMR (162 MHz, DMSO-*d_6_*) δ = 36.50 ppm. ^1^H NMR (400 MHz, DMSO-*d_6_*) δ = 2.68 (dd, 1H, *J* = 6.7, 0.9 Hz), 2.73 (dd, 1H, *J* = 6.7, 0.9 Hz), 5.99–6.11 (m, 1H), 6.62 (dd, 1H, *J* = 11.1, 4.9 Hz), 7.41 (d, 1H, *J* = 2.6 Hz), 7.66 (d, 1H, *J* = 2.6 Hz) ppm. ^13^C NMR (101 MHz, DMSO-*d_6_*) δ = 27.3 (d, *J*_P,C_ = 125 Hz), 126.1 (d, *J*_P,C_ = 12.2 Hz), 127.2 (d, *J*_P,C_ = 3.8 Hz), 127.5 (d, *J*_P,C_ = 8.6 Hz), 128.2, 128.5, 128.9, 131.2, 143.0 (d, *J*_P,C_ = 7.4 Hz) ppm. HRMS (ESI) [M + H]^+^: *m/z* calcd for C_9_H_8_O_3_PCl_2_: 264.9588, found 264.9595.

#### 2-Hydroxy-7-nitro-3H-benzo[f][1,2]oxaphosphepine 2-oxide (7 g)


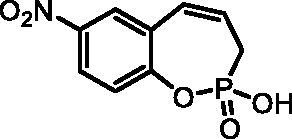
By following the general procedure, **7 g** was prepared from 2-ethoxy-7-nitro-3*H*-benzo[*f*][1,2]oxaphosphepine 2-oxide (**6 g**) (0.33 g, 1.23 mmol) and TMSBr (0.96 ml, 7.36 mmol) as a white solid (0.21 g, 71%). Mp: 183–184 °C. IR (KBr, cm^−1^): 2558 (O = P-OH), 2263 (O = P-OH), 1262 (P = O), 1221 (P = O). ^31^P NMR (162 MHz, DMSO-*d_6_*) δ = 39.26 ppm. ^1^H NMR (400 MHz, DMSO-*d_6_*) δ = 2.69 (dd, 1H, *J* = 6.6, 0.9 Hz), 2.74 (dd, 1H, *J* = 6.6, 0.9 Hz), 6.02–6.15 (m, 1H), 6.73–6.80 (m, 1H), 7.32–7.36 (m, 1H), 8.18 (dd, 1H, *J* = 8.9, 2.9 Hz), 8.25 (d, 1H, *J* = 2.9 Hz) ppm. ^13^C NMR (101 MHz, DMSO-*d_6_*) δ = 27.4 (d, *J*_P,C_ = 125 Hz), 123.2 (d, *J*_P,C_ = 3.6 Hz), 124.3, 126.0 (d, *J*_P,C_ = 12.0 Hz), 126.3, 127.7 (d, *J*_P,C_ = 9.0 Hz), 129.1, 143.7, 152.7 (d, *J*_P,C_ = 7.6 Hz) ppm. HRMS (ESI) [M + H]^+^: *m/z* calcd for C_9_H_9_NO_5_P: 242.0218, found 242.0226.

### Carbonic anhydrase inhibition assay

The CA-catalysed CO_2_ hydration activity was assayed by using an applied photophysics stopped-flow apparatus^24^. Phenol red (0.2 mM) was used as indicator following the initial rates of the CA-catalysed CO_2_ hydration reaction for a period of 10–100 s. The indicator worked at the absorbance maximum of 557 nm, with 20 mM HEPES buffer (pH 7.4) and 20 mM NaClO_4_ for maintaining constant ionic strength. For the determination of the kinetic parameters and inhibition constants, the CO_2_ concentrations were varied from 1.7 to 17 mM. For each inhibitor, at least six traces of the initial 5–10% of the reaction were used for determining the initial velocity. The uncatalysed rates were determined in the same fashion and subtracted from the total observed rates. The stock solutions of inhibitor were prepared as 1 mM solutions in distilled, deionised water. Afterwards, dilutions down to 0.01 nM were prepared in distilled and deionised water. Inhibitor and enzyme were preincubated together for 6 h at room temperature in order to allow for the formation of the enzyme–inhibitor complex. The inhibition constants were acquired by the non-linear least squares method using PRISM 3 and the Cheng–Prusoff equation, whereas the kinetic parameters of uninhibited enzymes were obtained from Lineweaver–Burk plots and represent the mean from at least three different determinations. All CA isoforms were recombinant, obtained in-house as reported earlier^25–27^.

## Results and discussion

### Chemistry

**Scheme 1.**
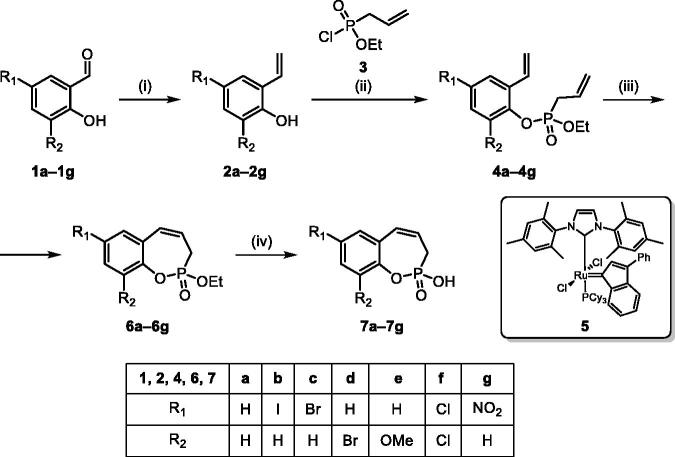
Reagents and conditions: (i) MePPh_3_Br, *t*-BuOK, THF, rt, 18 h, 76–88%; (ii) NEt_3_, DCM, 0 °C to rt, 18 h, 60–94%; (iii) **5**, PhMe, 70 °C, 4 h, 63–86%; (iv) TMSBr, DCM, rt, 24 h, 71–90%.

The synthetic strategy for the synthesis of 3*H*-1,2-benzoxaphosphepine 2-oxides is outlined in Scheme 1. The synthesis commenced with the Wittig reaction of commercially available 2-hydroxybenzaldehydes **1**, which provided olefins **2** in high yields. In the following step, compounds **2** were treated with ethyl allylphosphonochloridate (**3**, the reagent was prepared according to the literature procedure^23^) to give diolefins **4** in good to excellent yields. These key intermediates **4** were successfully cyclised by ring-closing metathesis, utilising commercially accessible Ru-based catalyst **5**. The reaction furnished corresponding cyclic ethyl phosphonates **6** in good yields. Finally, compounds **6** were treated with TMSBr to afford hydroxy derivatives **7** in very good yields.

### Carbonic anhydrase inhibition

The newly synthesised compounds **6** and **7** were evaluated for their CA inhibition activity by using the stopped-flow CO_2_ hydrase assay^24^. The study was carried out against four human CA isoforms – the ubiquitous cytosolic CA I and II as well as *trans*-membrane tumour-associated CA IX and XII^1–7^. The clinically used acetazolamide (AAZ) was used as the reference drug. The results of this study are shown in Table 1, and the following inferences could be drawn:

**Table 1.** Inhibition data of compounds **6–7** and the standard inhibitor acetazolamide (AAZ) against human CA isoforms I, II, IX and XII.

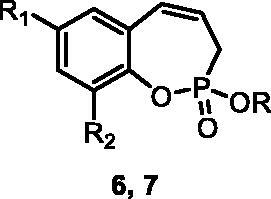							
Cmpd	R	R_1_	R_2_	*K*_I_ (µM)^a,b^			
				hCA I	hCA II	hCA IX	hCA XII
**6a**	Et	H	H	>100	>100	0.82	0.82
**7a**	H	H	H	>100	>100	1.3	0.51
**6b**	Et	I	H	>100	>100	4.7	2.4
**7b**	H	I	H	>100	>100	0.88	0.68
**6c**	Et	Br	H	>100	>100	0.76	1.6
**7c**	H	Br	H	>100	>100	1.0	0.96
**6d**	Et	H	Br	>100	>100	11.3	3.3
**7d**	H	H	Br	>100	>100	2.5	1.8
**6e**	Et	H	OMe	>100	>100	9.0	7.4
**7e**	H	H	OMe	>100	>100	1.8	1.2
**6f**	Et	Cl	Cl	>100	>100	6.1	3.4
**7f**	H	Cl	Cl	>100	>100	0.80	1.7
**6g**	Et	NO_2_	H	>100	>100	3.9	0.95
**7g**	H	NO_2_	H	>100	>100	0.67	1.0
**AAZ**	–			0.25	0.012	0.025	0.006

^a^Values are mean from three different assays using the stopped-flow technique (errors were in the range of ± 5–10% of the reported values).

^b^6 h incubation.

- All synthesised benzoxaphosphepine2-oxide derivatives **6–7** have no inhibitory activity towards cytosolic isoforms hCA I and hCA II (*K*_I_ > 100 µM), whose inhibition in most cases is undesirable, as hCA I and II isoforms are found in many tissues of the organism^1^^,^^2^^,^^7^. It should be mentioned that AAZ is a highly effective inhibitor of all the four hCA isoforms considered here, which explains the many side effects of that drug^28^^,^^29^.
- The tumour-associated hCA IX isoform was inhibited by all synthesised compounds **6–7** with inhibition constants in the sub-micromolar to low micromolar range (*K*_I_: 0.67–11.3 µM). The compound **7g** was found to be the most potent hCA IX inhibitor among tested compounds with *K*_I_ = 0.67 µM.
- The other tumour-associated isoform hCA XII was also notably inhibited by all the synthesised derivatives **6–7** with *K*_I_ values in the low micromolar and sub-micromolar range (*K*_I_: 0.51–7.4 µM). Among all tested compounds, compound **7a** was the most effective inhibitor against hCA XII with *K*_I_ = 0.51 µM.
- Overall, hydroxy derivatives **7** showed slightly higher inhibition potency against tumour-associated hCA IX and XII than the corresponding ethoxy derivatives **6**. In the case of hydroxy derivatives **7**, the range of *K*_I_ values was found to be from 0.67 to 2.5 µM for hCA IX and from 0.51 to 1.8 µM for hCA XII. Regarding ethoxy derivatives **6**, the range of *K*_I_ values was from 0.76 to 11.3 µM for hCA IX and from 0.95 to 7.4 µM for hCA XII.

Albeit the efficacy of the synthesised compounds **6–7** was lower in comparison to the reference drug AAZ, these compounds displayed desirable isoform-selective inhibition activity for tumour-associated isoforms hCA IX and hCA XII. The establishing of the selectivity is necessary to prevent possible side effects from inhibition of cytosolic hCA I and II isoforms^7^^,^^28^^,^^29^.

## Conclusions

Herein we report the synthesis of novel benzoxaphosphepine 2-oxide derivatives as a new class of tumour-associated CA IX and XII inhibitors. These compounds were investigated against four human CA isoforms with pharmacological applications (hCA I, hCA II, hCA IX, and hCA XII). All tested compounds exhibited selective inhibition of the tumour-associated hCA isoforms IX and XII with activities in the sub-micromolar or low micromolar range, whereas the off-target cytosolic isoforms hCA I and II were not significantly inhibited by these compounds. Considering that hCA IX and XII are implicated in processes connected to tumourigenesis^2–6^, present findings give an insight towards development of new selective anti-tumour agents.
